# RNA Silencing Sheds Light on the RNA World

**DOI:** 10.1371/journal.pbio.0040448

**Published:** 2006-12-05

**Authors:** Rachel Jones

RNA silencing — also known as RNA interference — is an intriguing phenomenon in which short, double-stranded RNA “triggers” can prevent the expression of specific genes. First discovered in plants, RNA interference is now recognized as a widespread, if not ubiquitous, phenomenon, and it is causing great excitement as an experimental technique for selectively blocking gene expression.

The mechanisms of RNA silencing have been intensively studied. One important step is the formation of single-stranded RNA pieces (called siRNAs) from the double-stranded triggers. In “lower” organisms—including plants, protozoa, fungi, and nematode worms—it also involves an enzyme—called RNA-dependent RNA polymerase—that can generate a strand of RNA using existing RNA as a template. This means that it can create double-stranded RNA from single-stranded pieces of RNA. By doing this, it generates more triggers and so amplifies the effect of RNA silencing. Paula Salgado and her colleagues have studied the structure of one such polymerase, called QDE-1, and found that it provides clues to the earliest stages of evolution.

When a gene is transcribed and translated to generate a protein, the process begins with a DNA-dependent RNA polymerase. Like QDE-1, DNA-dependent RNA polymerases generate strands of RNA—the difference is that they use a DNA template to do it. The RNA they generate is called messenger RNA and is in turn used as the template for building a protein out of amino acids. The structures of DNA-dependent RNA polymerases have been described previously, so that the authors of this study could compare them with their new structure of QDE-1.

What they found was a remarkable similarity. Both DNA-dependent RNA polymerases and QDE-1 have an active catalytic site—the working core of the enzyme—that is formed by two distinctive structural domains called double-psi β-barrels. This strong structural resemblance between QDE-1 and the DNA-dependent RNA polymerases points towards an evolutionary link between the two types of RNA polymerase.

An influential theory on the origin of life proposes that RNA molecules were the first self-replicating molecules, forming a kind of precellular life in an “RNA world.” Initially, RNA molecules would have had to act as enzymes as well as genetic information so that they could replicate, but it is likely that an RNA-dependent RNA polymerase would have been one of the earliest protein-based enzymes to evolve.

**Figure:**
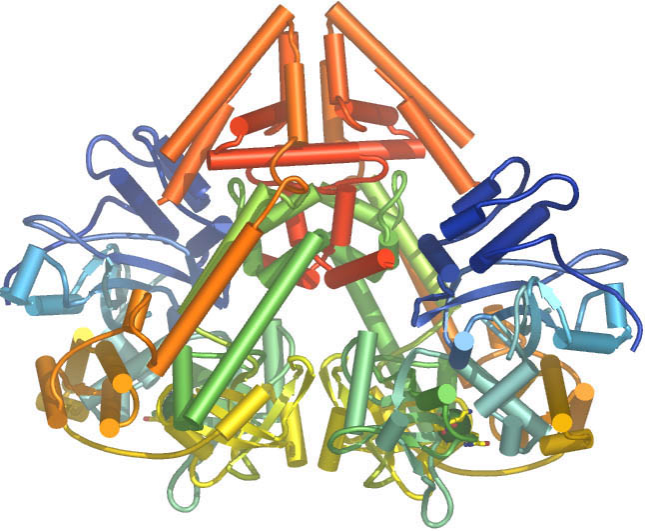
Schematic showing the fold of the QDE-1 RNA interference polymerase. The dimeric molecule is shown with the polypeptide chains colored from blue at the N termini to red at the C termini.

The similarity between QDE-1 and the DNA-dependent RNA polymerases suggests that both evolved from one ancestor, because otherwise, the resemblance between their active sites would be highly unlikely. This common ancestor might have been a primordial RNA polymerase in an RNA world. The authors suggest that this ancestor would have had just one double-psi β-barrel, and that gene duplication led to an enzyme with two barrels. From this early polymerase evolved both QDE-1–like RNA-dependent RNA polymerases and a diverse array of specialized DNA-dependent RNA polymerases. The diversification of DNA-dependent RNA polymerases would have been facilitated by the splitting of the two double-psi β-barrels onto separate subunits, rather than being borne on the same subunit as in QDE-1, so that different subunits could combine to create specialized polymerases.

These findings provide a link between RNA silencing and the earliest mechanisms of RNA transcription—perhaps shedding light on both the origins of RNA replication (and therefore life) and the evolution of RNA silencing. They also provide insights into the mechanism of action of QDE-1 that might apply across the board to RNA-dependent RNA polymerases, and that will be built upon by further work.

